# Putrescine-bound structure and multi-temperature crystallography provide new insights into ligand binding and conformational heterogeneity in spermidine synthase

**DOI:** 10.1063/4.0001217

**Published:** 2026-09-28

**Authors:** Omowumi O. Fagbohun, Molly A. Canfield, Jonathan A. Clinger

**Affiliations:** Department of Chemistry & Biochemistry, Baylor University, One Bear Place #97028, Waco, Texas 76798-7028, USA

## Abstract

Spermidine synthase (SPDS) is an important enzyme in the pathway for the biosynthesis of polyamines, and it synthesizes the polyamine spermidine from putrescine (PUT) and decarboxylated S-adenosylmethionine. This pathway is a promising target for antiproliferative therapies because of the functions of polyamines in cellular proliferation and differentiation. Higher polyamine levels are linked to cancer and cell proliferation. Previous structures of the enzyme were solved at cryogenic temperatures. Here, we report a new SPDS structure in complex with PUT (SPDS-PUT) and multitemperature crystallography of SPDS complexed with PUT and 5-deoxy-5-methylthioadenosine (MTA) at 100, 273, and 293 K to study its structure and dynamics. The results revealed that the occupancy of the active site is important for the extent of stabilization in the gatekeeping loop. The SPDS-PUT structure revealed putrescine binding in the absence of MTA, providing insight into its interactions within the active site and the conformational state of the gatekeeping loop. The binding of PUT alone was not enough to stabilize the gatekeeping loop, and greater stabilization of the loop occurred in the presence of MTA alone compared to PUT alone. The observed structural asymmetry between the gatekeeping loops of SPDS reflects differences in ligand binding between chains. These findings expand our understanding of the conformational landscape of SPDS.

## I. INTRODUCTION

Polyamines are molecules containing two or more amino groups that perform significant functions in cells.^1–5^ The charge distribution of polyamines promotes interaction with diverse negatively charged biomolecules such as proteins, nucleic acids, and acidic phospholipids.^5–7^ Polyamines are crucial for stabilizing DNA and RNA, playing vital roles in cellular development, stress responses, and regulation of genes, hence rendering them important in both pathological and physiological conditions.^1,6,8–11^ They perform pleiotropic roles in cellular processes such as redox equilibrium important for cell-to-cell communication, immunity, adhesion of cells, synthesis of proteins, proliferation, extracellular matrix repair, autophagy, and many types of cell death.^12–19^ Intracellular polyamine concentrations must be tightly controlled because of their pleiotropic role, ensuring a careful balance between transport, biosynthesis, and degradation. Accordingly, polyamine biosynthetic enzymes are tightly regulated at different levels, such as transcription, translation, and post-translational modification.^19^

The main pathway for the biosynthesis of polyamines involves the enzymes ornithine decarboxylase, spermidine synthase (SPDS), spermine synthase, and S-adenosylmethionine decarboxylase (AdoMetDC) and involves the decarboxylation of ornithine and aminopropyl transfer reactions to produce PUT, spermidine, and spermine.^5,20–22^ Given the essential roles of the polyamine biosynthetic pathway and its involvement in numerous diseases, this pathway is an important target for therapeutic research. The metabolism of polyamines is often dysregulated in cancer, suggesting that increased polyamine levels are essential for cellular transformation and tumor growth.^12,20,23^ It has also been demonstrated that spermidine is essential for malignant transformation, proliferation, and MYC (MYC proto-oncogene) expression.^24–26^ The dysregulation of polyamine metabolism has been implicated in several malignancies, including breast, colorectal, prostate, and skin cancers, highlighting the importance of the pathway as a therapeutic and chemopreventive target. Thus, polyamines and their metabolic derivatives are considered biomarkers of cancer.^5,27–31^

Spermidine synthase (SPDS) is an essential enzyme in the polyamine biosynthetic pathway, and it catalyzes the transfer of an aminopropyl group to putrescine (PUT) to produce spermidine, a polyamine, and 5-deoxy-5-methylthioadenosine (MTA) as a by-product. The catalytic residue, Asp173, deprotonates the N1 amine of PUT, thus performing a nucleophilic attack on the carbon next to the sulfonium center on the aminopropyl group. The reaction is also stabilized by the conserved amino acid residue, the backbone carbonyl of Ser174, and the hydroxyl groups of Tyr79 and Tyr241. The reaction is completed as the aminopropyl group is transferred to PUT and the electron pair gets transferred to the sulfur atom, producing spermidine and MTA (Fig. S1). The aminopropyl group from decarboxylated S-adenosylmethionine (dcAdoMet) is synthesized by AdoMetDC, which is a rate-limiting enzyme for the production of spermidine. Spermidine is one of the major polyamines essential for cell growth and development.^2,32–36^ It is involved in several biological roles, including suppressing nitric oxide, modulating membrane potential, and stimulating autophagy. Also, the elF5A initiation factor requires an aminobutyl group from spermidine for its posttranslational modification. The first process relies on spermidine, which donates the 4-aminobutyl group that is intended to bind to eIF5A's lysine amino group, thus generating deoxyhypusine, an intermediate. Subsequently, hypusine is generated by the hydroxylation of the intermediate.^37–40^

The structure of human SPDS is a homodimer composed of the N-terminal domain, the central core domain, and the C-terminal domain (Fig. 1). The dimerization interface is formed by the N-terminal and C-terminal domains, and the active site is in between the N-terminal and central core domains, consisting of both the dcAdoMet and PUT binding pockets.^2,34,36^ The gatekeeping loop contains a short alpha helix, and it is positioned near the active site entrance, enclosing the catalytic pockets.^2,32,34,36^ This flexible loop is completely disordered in apo human SPDS, as in other apo homologous SPDS structures.^2,4,32,41–44^ However, it is ordered in ligand-bound structures. The loop is also stabilized in the presence of PUT, as demonstrated by the comparison of the structure of the SPDS-MTA complex with that of SPDS-MTA-PUT.^36^

**FIG. 1.**
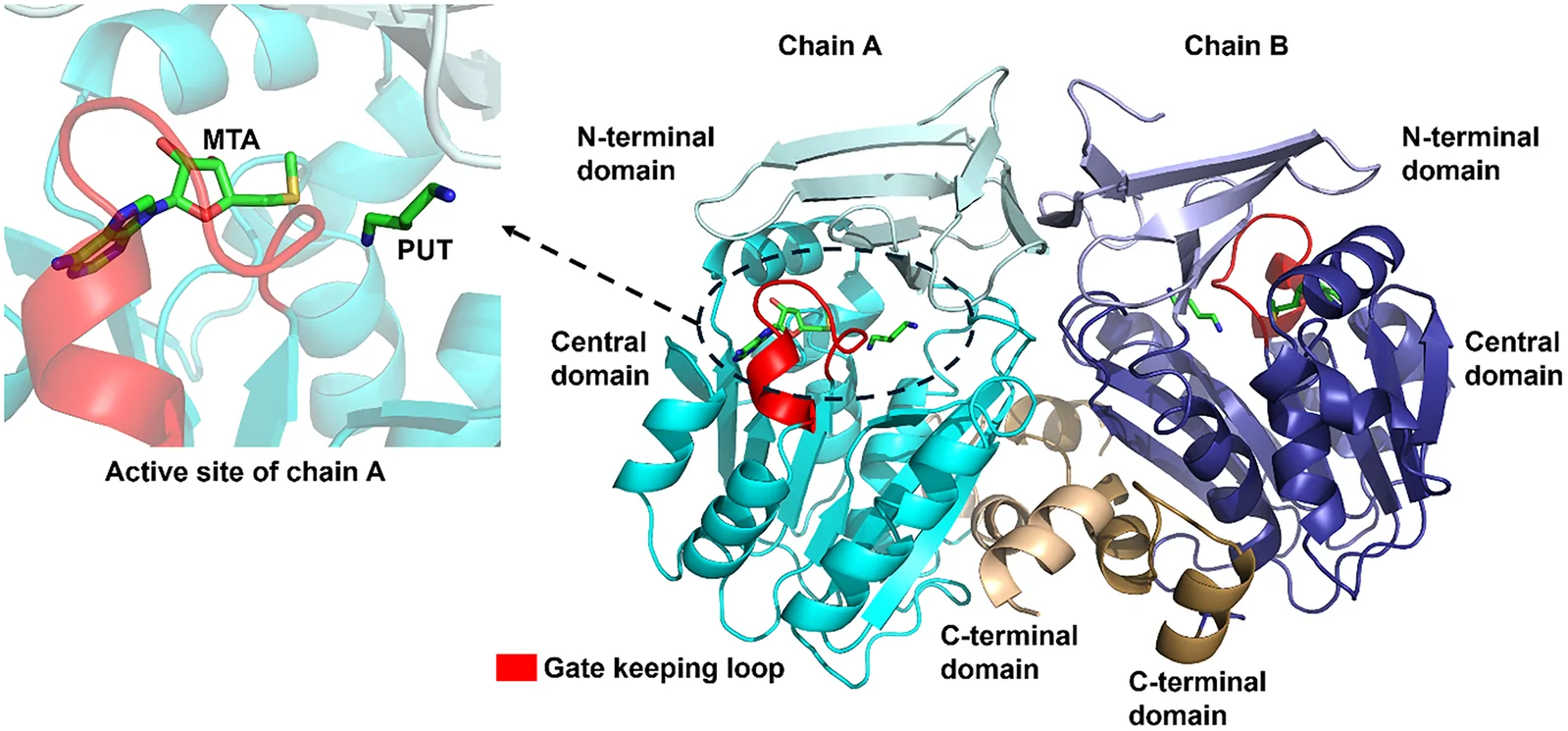
Structural overview of SPDS. SPDS consists of two chains, each containing N-terminal, central core, and C-terminal domains. The active site is located between the N-terminal and central core domains and contains the putrescine and aminopropyl donor binding pockets (green) and a gatekeeping loop (red).

Multitemperature crystallography of SPDS was performed to probe the structural transitions of the enzyme at different temperatures, 100, 273, and 293 K. At cryogenic temperatures, conformations of the side and main chains of enzymes have been reported to be hidden or altered.^45–49^ Previously unknown conformations have been discovered through crystal structure determination at room temperature. Regions of the protein were observed to change from having one conformation to several conformations with an increase in temperature.^48,50–58^ The structures of AdoMetDC collected at ambient temperature showed a different conformation of the DL2 (disordered loop 2) region compared to the structure determined at cryogenic temperature.^21^ Thus, the structure of SPDS was solved at 100, 273, and 293 K to study the temperature dependence of the enzyme. The multitemperature structures allowed for investigation of possible asymmetry between the two chains of SPDS. Multiple analyses on the multitemperature structures were carried out to investigate temperature-dependent conformational changes. The structure of the SPDS-PUT complex was also solved to determine the specific role of PUT in stabilizing the gatekeeping loop in the absence of MTA or dcAdoMet. The average B-factor of the loop in the SPDS-PUT structure was compared to the average B-factors of the corresponding region in previously deposited human SPDS structures. These results improved our knowledge of the conformational changes in SPDS at near physiological temperatures and in the absence of the aminopropyl donor.

## II. METHODS

### A. Expression and purification

The plasmid pET-27b(+) containing SPDS gene (human) with 6× His-tag and Tobacco Etch Virus (TEV) cleavage site at the N-terminus was purchased from GenScript. Transformation of the plasmid DNA was performed using chemically competent *Escherichia coli* BL21 cells obtained from New England Biolabs. The cells were cultured at 37 °C to OD600 between 0.6 and 0.8 with 50 *μ*g/ml of kanamycin in 2 × YT media and induced for SPDS expression using 1 mM IPTG (isopropyl b-D-1-thiogalactopyranoside). Post induction, the cells were incubated overnight at 15 °C and thereafter centrifuged at 6000 rpm for 45 min. The cell pellets were harvested and stored overnight at −80 °C. Resuspension of pellets was performed using a buffer containing 250 mM sodium chloride, 100 mM HEPES (2-[4-(2-hydroxyethyl) piperazin-1-yl] ethane sulfonic acid) pH 7.5, and 5 mM imidazole. The cells were lysed using a sonicator for 20 min with 20 s intermittent off pulses and subsequently centrifuged for 30 min at 21 000 rpm. The supernatant was collected and purified using a HisTrap HP Ni-NTA column (Cytiva). The running buffer remained the same as the resuspension buffer, but the elution buffer contained a higher concentration of imidazole of 250 mM. Further purification of the protein was performed using a Superdex 200 PG size-exclusion column (HiLoad 16/600, Cytiva). The buffer contained 200 mM sodium chloride and 25 mM HEPES pH 7.5.

### B. Crystallization

Human SPDS with MTA and PUT was crystallized using the sitting-drop vapor diffusion method. The crystallization buffer contained 6.5%–23% polyethylene glycol 3350, 0.1 M Bis-Tris buffer, pH 5.5, and 0.05–0.225 M sodium sulfate. A concentration of 10 mg/ml of SPDS was used for crystallization, and the mixture of SPDS with MTA and PUT contained a 1:5:10 molar ratio of SPDS:MTA:PUT. SPDS with PUT was crystallized in a 1:10 molar ratio of SPDS:PUT and with a very low concentration of MTA (0.34 mM in a 1:1.13 molar ratio of SPDS to MTA). In the crystallization tray, the bottom wells contained 100 *μ*l of mother liquor, and the top wells contained a 1:1 v/v mixture of SPDS-MTA-PUT and mother liquor. The tray was placed at room temperature, and SPDS crystals grew within 24 h. For the 100 K data, crystals were cryoprotected with 10% glycerol and plunged into liquid nitrogen. The 273 and 293 K data were collected directly at the Stanford Synchrotron Radiation Lightsource (SSRL) by adjusting the cryo-stream temperature to 273 and 293 K, respectively, before mounting the crystals. These crystals were covered with RT tubes (RT-T1 MicroRT Tubes purchased from MiTeGen) containing the mother liquor and were not cryoprotected. For SPDS-PUT data, crystals were cryoprotected with 10% glycerol and cryocooled in the cryo-stream at SSRL.

### C. Data collection, processing, and refinement

Diffraction data were collected at beamline 12-1 at SSRL. SSRL auto-processing pipeline (autoPROC) was used to process data, which incorporates XDS for data reduction, POINTLESS to assign the space group, and AIMLESS for scaling/merging datasets.^59–63^ The AIMLESS file from autoPROC was used for Phenix (version 1.21.2-5419-000) downstream processing.^64^ For SPDS-PUT, data processing was carried out using DIALS within the automated xia2 pipeline,^65^ and POINTLESS and AIMLESS were used for space group assignment, scaling, and merging.^61^ To assess data quality and ensure the absence of warnings, analysis by Xtriage was performed. SPDS-PUT and 293 K datasets were solved using PDB 2O07^36^ as the starting structure on Phenix.refine, and the refined 293 K dataset was subsequently used to refine the 273 and 100 K datasets. R-free flags were generated for the 100 K data and SPDS-PUT data using FREERFLAG (a CCP4-supported program) to ensure 5% R-free flags were used. The generated 100 K R-free flags were transferred to 273 and 293 K, datasets using the Phenix reflection editor for consistency across all multitemperature datasets.^66,67^ COOT was used for manual model building (version 0.9.8.95).^68^ The 100, 273, and 293 K SPDS-MTA-PUT datasets were processed to 1.71, 1.98, and 1.94 Å resolution, respectively. SPDS-MTA structure was determined at 1.76 Å resolution. The data collection statistics are shown in Table I.

**TABLE I.** X-ray diffraction data collection, processing, and refinement statistics.

	100 K	273 K	293 K	SPDS-PUT
PDB ID	13FV	13FW	13FX	13HN
Wavelength (Å)	0.979 46	0.979 46	0.979 46	0.979 46
Resolution range (Å)	54.87–1.71 (1.80–1.71)	54.36–1.98 (2.03–1.98)	82.38–1.94 (2.04–1.94)	48.86–1.76 (1.79–1.76)
Space group	P 1 21 1	P 1 21 1	P 1 21 1	P 1 21 1
Unit cell [a, b, c (Å) α, β, γ (°)]	57.977 60.812 86.443 90 108.85 90	57.332 61.265 87.239 90 108.51 90	58.974 61.376 86.907 90 108.57 90	58.208 60.725 86.674 90 108.36 90
Total reflections	201 331 (29 242)	93 803 (6670)	117 019 (17 441)	182 123 (10 456)
Unique reflections	61 324 (8948)	38 154 (2696)	42 266 (6321)	55 432 (3177)
Multiplicity	3.3 (3.3)	2.5 (2.5)	2.8 (2.8)	3.3 (3.3)
Completeness (%)	98.8 (99.1)	95.3 (96.7)	96.9 (99.3)	97.3 (97.8)
Mean I/sigma(I)	6.1 (1.8)	4.2 (1.5)	3.9 (1)	5.4 (1.9)
Wilson B-factor	15.71	18.2	19.4	15.2
R-merge	0.125 (0.950)	0.179 (1.171)	0.149 (0.891)	0.135 (0.716)
R-meas	0.150 (1.132)	0.221 (1.430)	0.184 (1.104)	0.163 (0.861)
R-pim	0.081 (0.608)	0.127 (0.809)	0.106 (0.644)	0.089 (0.470)
CC1/2	0.99 (0.590)	0.981 (0.531)	0.985 (0.457)	0.948 (0.447)
Reflections used in refinement	58 082 (2505)	36 179 (2602)	40 037 (2711)	52 520 (2627)
Reflections used for R-free	3207 (141)	1941 (122)	2153 (157)	2758 (151)
R-work	0.1706 (0.2746)	0.1737 (0.2630)	0.1655 (0.2618)	0.1796 (0.2530)
R-free	0.2099 (0.3156)	0.2221 (0.3134)	0.2066 (0.2878)	0.2253 (0.3252)
Number of non-hydrogen atoms	5125	4934	4956	4936
Macromolecules	4521	4472	4524	4439
Ligands	57	52	52	6
Solvent	547	410	380	491
Protein residues	571	570	571	561
RMS [bonds (Å)]	0.006	0.004	0.006	0.011
RMS [angles (°)]	0.82	0.86	0.88	0.93
Ramachandran favored (%)	97.18	97	97.53	97.12
Ramachandran allowed (%)	2.82	3	2.47	2.88
Ramachandran outliers (%)	0	0	0	0
Rotamer outliers (%)	0	0	0	0.2
Clashscore	2.1	1.01	1.1	2.95
Average B-factor (Å^2^)	22.4	22.25	24.15	20.15
Macromolecules	19.78	21.53	23.47	19.30
Ligands	24.33	23.25	27.22	40.22
Solvent	30.17	30.02	31.80	27.51

MTA was modeled into the active site of the SPDS-PUT structure and refined; however, the refined model showed poor agreement with experimental data (Fig. S2). The B-factors of both MTA and PUT were adjusted to match the B-factors of the neighboring residues and re-refined. The result indicated that MTA was not present in the crystal structure. In the SPDS-PUT structure, PUT occupancy was refined by first adjusting its B-factors to match those of the surrounding residues and assigning an initial occupancy of 0.5 in Coot. Occupancy refinement was then performed on Phenix by refining occupancy only. A polder omit map was also calculated using the phenix.polder on Phenix. PUT was omitted from the refined model prior to map calculation, and the resulting mFo-DFc polder map was used to assess unbiased electron density supporting ligand placement (Fig. S3). The multitemperature structures, 100, 273, and 293 K was deposited in the Protein Data Bank with PDB IDs 13FV, 13FW, and 13FX, respectively. SPDS-PUT structure was also deposited with PDB ID 13HN. Diffraction images were deposited on SBGrid Data Bank with the following DOIs: 100 K, doi:10.15785/SBGRID/1264; 273 K, doi:10.15785/SBGRID/1265; 293 K, doi:10.15785/SBGRID/1266; and SPDS-PUT, doi:10.15785/SBGRID/1268.

### D. Structural analysis

Structural analysis of the multitemperature data was performed using normalized/absolute B-factors, Ringer and Flipper, the distance difference matrix (DDM), and RoPE (representation of protein entities). To compare the atomic displacement across multiple datasets, the average B-factors for all residues were normalized independently for each structure using Z-scores to account for differences in dataset quality. For each dataset, the mean residue-average B-factor was subtracted from each residue-average B-factor and divided by the standard deviation of the mean residue-average B-factor,

$$Z\_\text{score}=\frac{Bi-\text{avg}\_B}{\text{std}},$$ (1)

where Bi is the average B-factor of residue i, avg_B is the mean residue-average B-factor for the structure, and std is the corresponding standard deviation. This normalization removes global differences in overall B-factors between datasets while preserving relative differences in residue flexibility within each structure. For comparison of loop B-factors between chains A and B within each structure (Table II), absolute average B-factors were used because both chains were refined together and therefore share the same overall B-factor scaling.

**TABLE II.** Average B-factors for loop residues for new multitemperature structures and previously deposited structures.

	Average B-factors (Å^2^) of the loop (residues 174–185)			
Structures	Chain A	Chain B	Space group	Ligands
100 K	43.4	29.8	P12_1_1	MTA, PUT
273 K	43.4	37.2	P12_1_1	MTA, PUT
293 K	55	41.2	P12_1_1	MTA, PUT
2O07	Not modeled	31.4	P12_1_1	MTA, SPD
2O07_PDB_REDO	45.6	34.2	P12_1_1	MTA, SPD
2O06	25.8	39.8	C222_1_	MTA, PUT
SPDS-PUT	Not modeled	74	P12_1_1	PUT
2O05	43.1	41.2	P12_1_1	MTA
2O0L^36^	Not modeled	33	P12_1_1	DcAdoMet
2O0L_PDB_REDO	56.2	56	P12_1_1	DcAdoMet
9Q41	Not modeled	Not modeled	C222_1_	apo

Ringer and Flipper analyses were used for the detection of side chain rotamers.^57,69^ Ringer can detect low occupancy alternative side chain conformations, enabling comparison of side chain conformations across all multitemperature datasets. Ringer analysis was performed with *mmtbx.ringer* command using the PDB model and the corresponding map coefficients (map_coeffs.mtz). Map coefficients were generated from the refined model and the reflection file using *phenix.maps.* Ringer sampled electron density around the side chain dihedral angles from the 2mFo-DFo and mFo-DFo maps using a sigma cutoff of 0.5. The resulting Ringer outputs were analyzed using Flipper by comparing the cryogenic structure with the ambient temperature structures. Flipper is an extension of the Ringer algorithm, and it was accessed from https://github.com/TheFischerLab/Flipper,^57^ allowing visualization of changes in side chain conformations, including rotamer flips and conformer gains and losses, generating residue-specific plots and summary CSV files. A color-coded barcode plot of peak gains and losses was generated using Matplotlib.

The distance difference matrix (DDM) was used to compare the distances between Cα atoms.^70,71^ DDM analysis was performed using the ddm.py script accessed from https://github.com/malitharatnaonc/differencedistance. Pairwise Cα–Cα distances were calculated, and the differences between corresponding inter-residue distances were computed to generate DDMs for each pair of temperature datasets. Lower temperature datasets were subtracted from higher temperature datasets (D293_/273K_–D_100K_ and D_293_–D_273K_). Positive DDM values indicate larger Cα–Cα distances for higher temperatures relative to the reference dataset, and shorter Cα–Cα distances are indicated by negative DDM values. The resulting matrices were exported as CSV files and visualized as heatmaps using Matplotlib.

Torsional space comparison was performed using RoPE,^72^ accessed from https://rope.hginn.co.uk/index.php?page=install. Newly deposited structures, 100, 273, 293 K, and SPDS-PUT, together with previously deposited structures, apoSPDS (9Q41),^2^ SPDS-MTA-PUT (2O06), SPDS-MTA-spermidine (SPDS-MTA-SPD) (2O07), SPDS-dcAdoMet (2O0L), SPDS-MTA (2O05),^36^ and SPDS-decarboxylated S-adenosylhomocysteine (SPDS-dcSAH),^34^ were compared based on their backbone and side chain torsion angle distributions.

## III. RESULTS/DISCUSSION

The structure of human SPDS in complex with PUT was determined at a resolution of 1.76 Å. There is no electron density for MTA in this structure due to the low concentration of MTA used (Fig. S2). PUT was initially modeled in both chains; however, chain A has a much lower PUT occupancy (refined occupancy of 0.71) compared to chain B (refined occupancy of 0.84), which agrees with the calculated RSCC values of 0.55 and 0.79, respectively. A polder omit map was calculated to further validate the presence of PUT in the SPDS-PUT structure, which shows a well-defined electron density for PUT only in chain B, while the density in chain A was insufficient to confidently model PUT (Fig. S3). Therefore, PUT was removed from chain A in the final model. Comparison of the structure of the SPDS-PUT complex with the apo SPDS and other ligand-bound SPDS complexes revealed the role of ligand binding in stabilizing the gatekeeping loop. The gatekeeping loop in the structure of SPDS-PUT is highly disordered, indicating high flexibility in the absence of MTA. In a previous report, the binding of PUT stabilizes the gatekeeping as shown by the comparison of the average B-factors of the gatekeeping loop in the SPDS-MTA complex (41–43 Å^2^), with SPDS-MTA-PUT complex (28 Å^2^) and SPDS-MTA-spermidine (29 Å^2^).^36^ There is a lower B-factor in the SPDS-MTA-PUT structure compared to the SPDS-MTA structure, consistent with increased stabilization of the gatekeeping loop upon PUT binding. The normalized loop B-factors for these structures are 2.61 (chain A) and 2.44 (chain B) for SPDS-MTA, 0.57 (chain A) and 2.55 (chain B) for SPDS-MTA-PUT, and 2.67 (chain A) and 1.21 (chain B) for SPDS-MTA-SPD. The normalized average B-factor of this loop in the newly reported SPDS-PUT structure is 4.8 (74 Å^2^ absolute) in chain B, with no electron density for this loop in chain A, denoting the importance of MTA in stabilizing the gatekeeping loop. The SPDS-PUT structure showed a higher normalized average B-factor of the loop relative to the SPDS-MTA structure, demonstrating increased conformational flexibility. In the structure of apo SPDS (9Q41),^2^ the gatekeeping loop is completely disordered and therefore was not modeled, and the Asp173 catalytic residue was in an inactive conformation.^2^ In SPDS-PUT, the gatekeeping loop is less disordered relative to the apo structure in chain B, and the Asp173 residue is in the active conformation like other ligand-bound structures in both chains (Fig. S4). This suggests that PUT may partially occupy chain A, although the weak density did not support modeling PUT in this chain. Compared to the apo structure with the absence of electron density for the loop indicating complete disorder,^2^ the gatekeeping loop in the presence of either ligand is more stabilized and less flexible.

PUT interacts with Asp173 through its N1 amine, positioning it for nucleophilic attack, while Asp176 forms a hydrogen bond with the N4 amine of PUT in the SPDS-MTA-PUT (2O06) structure (Fig. 2).^36^ In the SPDS-MTA-SPD (2O07) structure, spermidine occupies the same binding pocket as PUT; however, the N1 amine adopts a bent conformation, whereas the N8 amine retains interaction with Asp176.^36^ In the SPDS-PUT structure, Asp173 forms a hydrogen bond with PUT at the N1 amine; however, there is no observed interaction between Asp176 and the N4 amine of PUT because the side chain of Asp176 lacks interpretable electron density. Consequently, the N4 amine in the SPDS-PUT structure is less constrained compared to the SPDS-MTA-PUT structure. In the SPDS-MTA structure, PUT is absent. Asp173 maintains the same active conformation, whereas Asp176 adopts a different side chain conformation in the absence of the Asp176-PUT hydrogen bond (Figs. 2 and S5). The differences in ligand interactions are accompanied by changes in the flexibility of the gatekeeping loop. The binding of both ligands contributes to the stability of the loop, with MTA responsible for much conformational order, while PUT alone does not stabilize the gatekeeping loop. Overall, the stability of the gatekeeping loop depends on the occupancy of the active site.

**FIG. 2.**
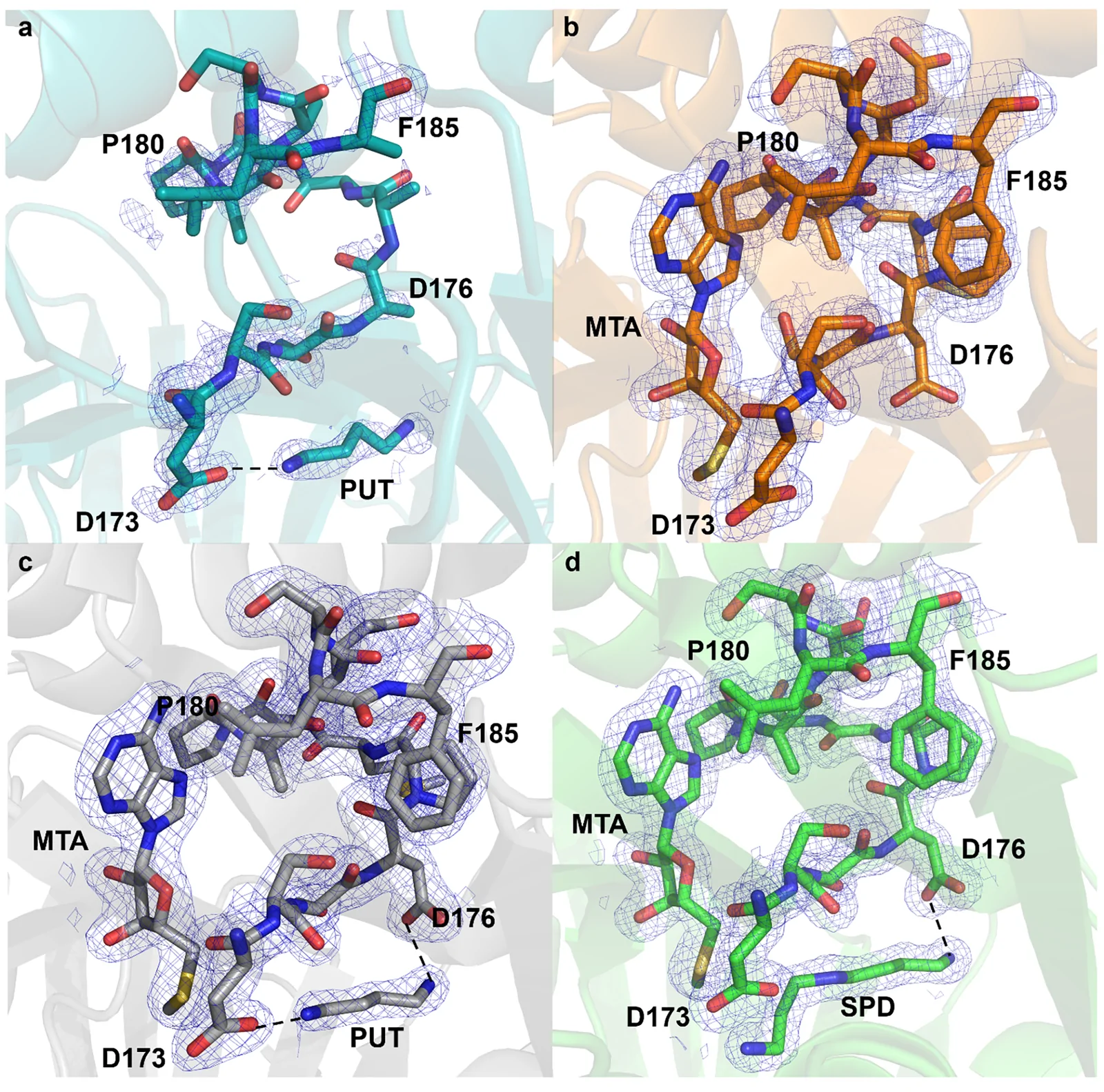
Interactions of PUT with Asp173 and Asp176, and the gatekeeping loop (residues 174–185) in (a) SPDS-PUT, (b) SPDS-MTA (2O05), (c) SPDS-MTA-PUT (2O06), and (d) SPDS-MTA-SPD (2O07) structures. The 2mFo-DFc map is shown in blue, contoured at an RMSD of 1.

To evaluate the differences between the multitemperature structures and the two chains of SPDS, the B-factors of the gatekeeping loop were compared (Figs. 3 and 4, and Table II). This loop exhibits different levels of conformational flexibility between the two chains across all structures. The gatekeeping loop in one chain of all structures consistently has a more ordered electron density compared to the corresponding region in the other chain. This is accompanied by lower average B-factors of the residues in this loop, indicating reduced conformational flexibility (Fig. 3 and Table II). The same trend is observed using normalized B-factors (Z scores), which accounts for differences in overall B-factor distributions between datasets (Fig. 4). In the loop region (residues 174–185), chain A consistently exhibits higher average normalized B-factors/absolute average B-factors than chain B at all temperatures (100 K: 2.44 vs 1.33 normalized, 43.4 vs 29.8 absolute; 273 K: 1.88 vs 1.12 normalized, 43.6 vs 37.2 absolute; 293 K: 2.24 vs 1.60 normalized, 55 vs 41.2 absolute), indicating greater relative flexibility of chain A. Notably, high average B-factors/normalized B-factors in the gatekeeping loop of one of the chains correlates with reduced PUT occupancy in this chain. This is consistent with the observations in the SPDS-PUT structure, where there is higher occupancy of PUT in one chain than the other. This further supports that ligand binding and occupancy influence the flexibility of the gatekeeping loop.

**FIG. 3.**
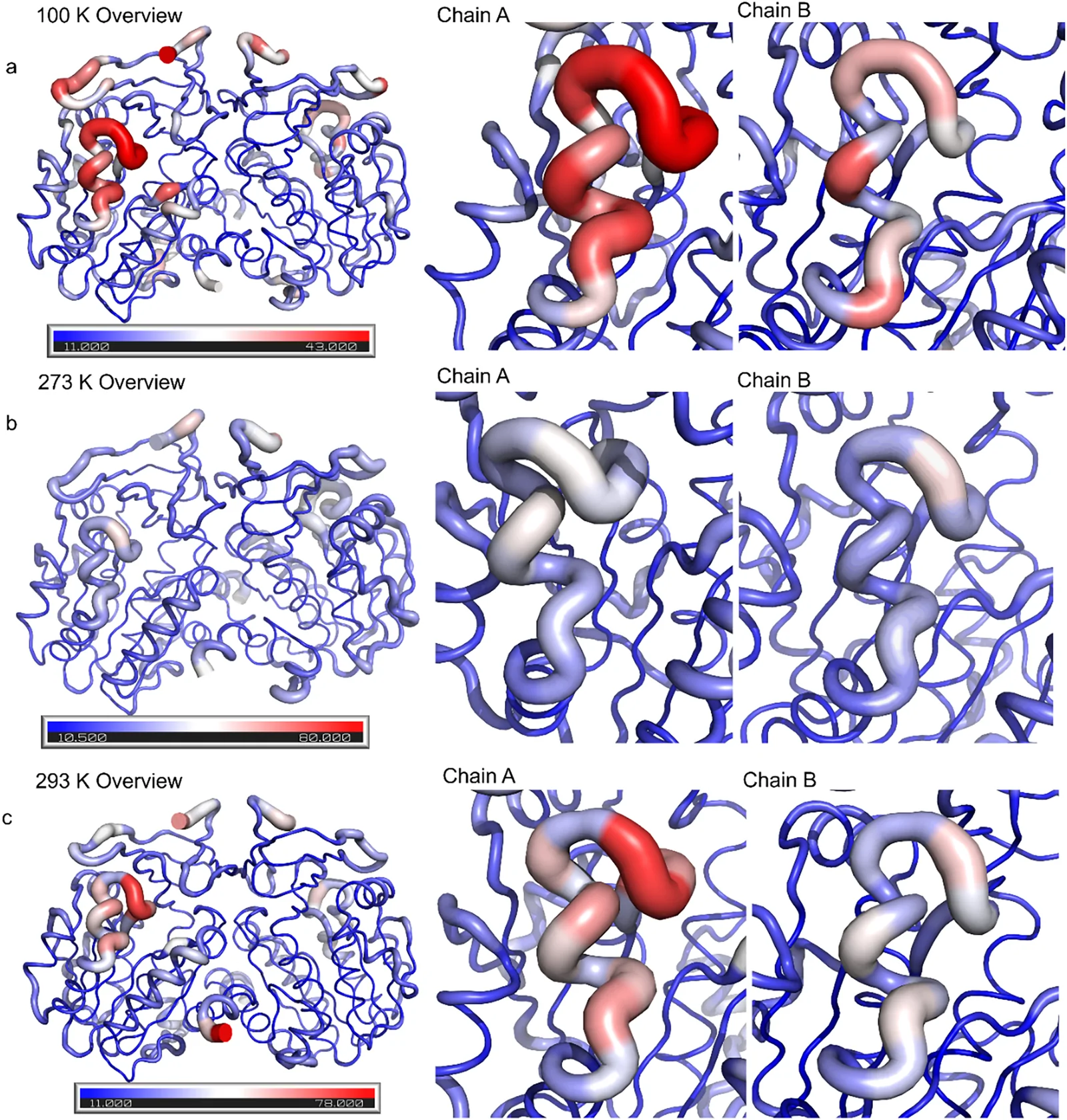
B-factor putty representation of SPDS at (a) 100 K (scale: 11–43), (b) 273 K (scale: 10.5–80), and (c) 293 K (scale: 11–78), showing differences in gatekeeping loop flexibility between chains A and B. Chain A consistently shows higher B-factors than chain B in the gatekeeping loop region across the three temperatures, illustrating the differences in structural flexibility.

**FIG. 4.**
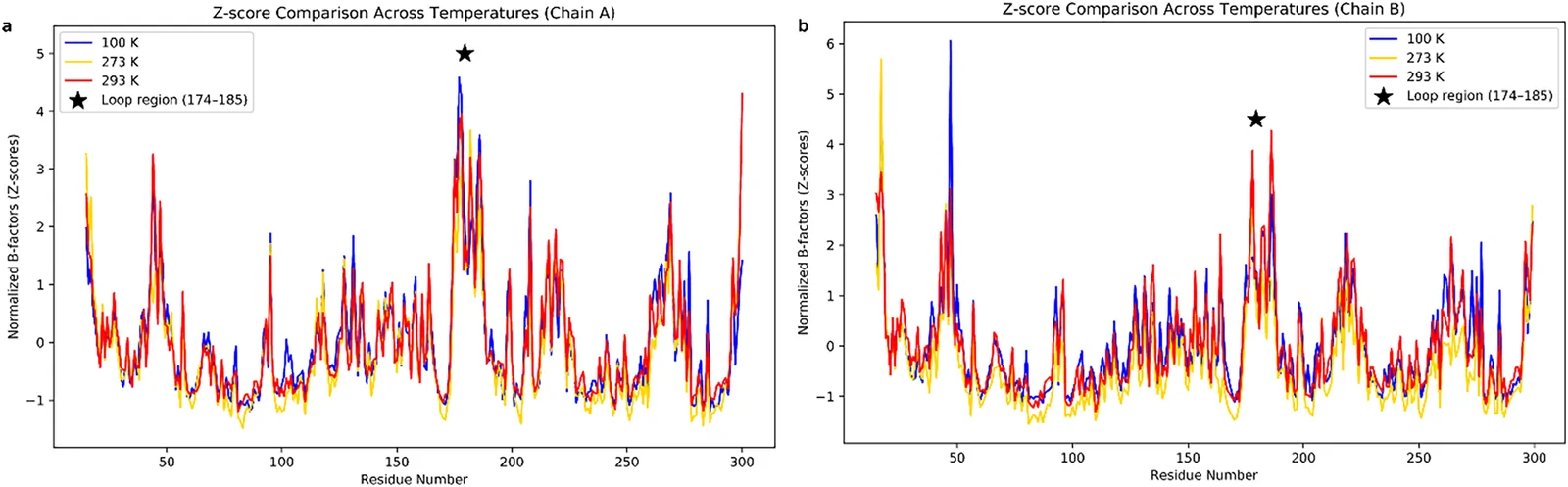
Normalized B-factor plots of 100, 273, and 293 K Å structures. (a) Chain A. (b) Chain B. The star represents the gatekeeping loop region.

This same pattern of higher average B-factor/conformational flexibility in the gatekeeping loop of one chain relative to the other exists in the previously deposited structure of SPDS-MTA-SPD (PDB 2O07), which has the same space group (P12_1_1) as the new structures (100, 273, and 293 K). Also, the previously deposited structure of SPDS-MTA-PUT (PDB 2O06)^36^ with a different space group (C222_1_) from the new structures has the same pattern. The catalytic residues (Asp173 and Ser174) that precede the disordered region in the gatekeeping loop in the apo human structure with space group C222_1_ were shown to have well-defined electron density in one chain compared to the other chain.^2^ In other words, this trend is observed in structures even with different space groups, with one chain consistently having a higher average loop B-factor than the other. No crystal contacts were observed near the gatekeeping loop in either chain, indicating that crystal contacts do not account for observed asymmetry (Fig. S6). The difference in flexibility of the gatekeeping loop between the two chains becomes more pronounced upon PUT binding, whereas in the absence of PUT, with only aminopropyl/MTA bound, both chains show comparable average loop B-factors. Structural asymmetry between protomers is increasingly recognized in homo-oligomeric proteins such as fluoroacetate dehalogenase, ribonucleotide reductases (RNR), and tumor necrosis factor receptor associated protein 1 (TRAP1), where the distinct conformational states of individual protomers have been linked to enzyme dynamics and catalytic regulations.^73–80^ Structural asymmetry was proposed to be induced by the simultaneous dimerization of the N-terminal and C-terminal domains in the homodimeric protein mitochondrial heat shock protein 90 (TRAP1), which leads to an asymmetric helix swap at the middle to the C-terminal domain.^76^ Asymmetry in thiamine-dependent enzymes and other homo-oligomeric enzymes, RNR and Tryptophan 2,3-dioxygenase (TDO), has been linked to distinctive catalytic characteristics, including half-of-sites reactivity and negative cooperativity.^73,80^ The asymmetric behavior of the gatekeeping loop between both chains of SPDS, with one consistently more ordered, suggests preferential stabilization of one active site at a time.

The 100, 273, and 293 K structures were determined to a resolution of 1.71, 1.98, and 1.94 Å, respectively. The differences between multitemperature structures of SPDS were subtle; thus, Ringer and Flipper, RoPE, and DDM were used to analyze the temperature differences between the data. Ringer and Flipper analyses were used to examine the side chain conformational heterogeneity^55,57,69^ at different temperatures (Figs. 5, S7, S8, and S9). The barcode plots of chi1 and chi2 show several residues with peak gains across the structures [Figs. 3(a) and 3(b)]. The results show a similar number of peak gains when the 100 K structure was compared with both the 273 K (51 vs 47 peak gains) and 293 K structures (49 and 42 peak gains) (Fig. S7). This indicates that some residues only exhibit alternate rotameric conformation at certain temperatures, and that overall heterogeneity of side chains is not increased between cryogenic and ambient temperatures. Examples of residues with alternate rotameric side chains that are detectable at cryogenic temperature are Cys205, Gln191, Ser281, and Glu208 (Fig. S8), and those that are detectable only at ambient temperatures are Ser256, Gln264, Glu158, and Ser145 (Fig. S9). The side chain alternate conformation of Ser256 is present in both chain A and B of the 273 K structures and chain A of the 293 K structure. At 100 K, the single side chain conformation of Glu158 is consistent in both chain A and chain B, while at 293 K, the conformational landscape of the residue broadens between both chains, suggesting asymmetric conformational sampling between the chains. Some residues show a shift in rotamer population at higher temperatures, such as Ser78, consistent in chain A and chain B of the 293 K structure and chain A of the 273 K structure [Fig. 5(c)]. The catalytic residue, Gln80, that interacts with the dcAdoMet aminopropyl group also appears to have an alternate side chain conformation above 0.5 sigma in both 273 and 293 K structures [Fig. 5(d)]. This alternative species would be unable to form a hydrogen bond with the amino group in dcAdoMet in this orientation. These observations reveal the heterogeneous temperature-dependent conformational sampling and redistribution of side chain rotamer populations at different temperatures, expanding our understanding of the conformational landscape beyond cryogenic temperatures.

**FIG. 5.**
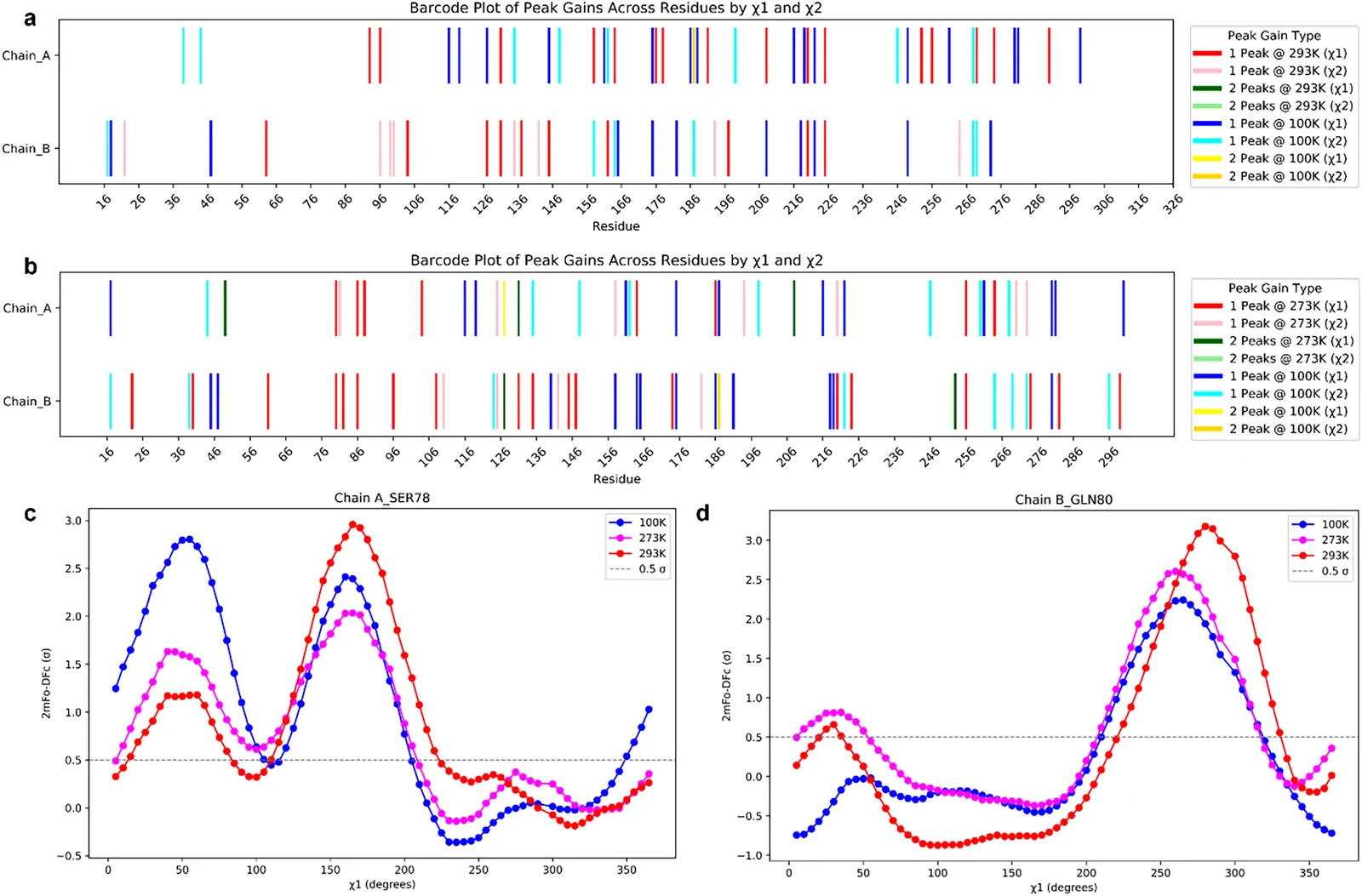
Temperature-dependent side chain conformational sampling by Flipper and Ringer analyses. (a) and (b) Flipper barcode plots of peak gains across residues in chains A and B for (a) 293 K and (b) 273 K relative to 100 K. Color-coded lines represent electron density peaks for chi 1 and chi 2 angles. (c) and (d) Representative Ringer plots showing the 2mFo-DFc map for the side chain rotamer population of (c) Ser78 and (d) Gln80.

DDM was used to determine the conformational changes between cryogenic and ambient temperature structures. The distance difference matrices between the Cα atoms were calculated by subtracting the 100 K structure from both the 273 and 293 K structures, and the 273 K from the 293 K structure. The results show a small and widely dispersed positive signal indicating increased atomic distance in the 273 and 293 K structures with an average distance difference of 0.08 and 0.13 Å for chain A and B in the 273_100 K comparison, and 0.1 and 0.11 Å in the 293_100 K comparison (Fig. 6). In the 273_293 K DDM comparison, the average distance difference in chain A is 0.01 and in chain B is −0.02, indicating minimal backbone arrangement between the two ambient temperature structures. This suggests a global expansion of the protein as it warms up and contraction of the protein by cryocooling, which is consistent with the measured unit cell parameters (Table I). Some localized regions contract as the protein warms up, as indicated by minor blue regions from negative signals. The expansion of the protein at ambient temperature is also not uniform; residues 186–188, which have the largest deviations in the structure, are right after the gatekeeping loop region, with an average distance ranging from 0.39 to 0.68 Å [Fig. 6(g)]. This indicates an increased displacement in some specific Cα atoms at higher temperatures. The largest distance differences in the 273_100 K DDM comparison for this region correspond to residue E188 in chain A (0.38 Å) and residue E186 in chain B (0.55 Å), which are lower than the largest distance differences observed in the 293_100 K DDM comparison for this region, corresponding to residue E187 (0.56 Å in chain A and 0.68 Å in chain B). This is consistent with the 293_273 K DDM comparison, in which the largest distance differences correspond to residue E187 (0.2 Å in chain A and 0.4 Å in chain B), indicating larger distance differences in this region for the 293 K structure. These results indicate that the helix is a component of loop opening, and at elevated temperatures, favors an expanded conformation. There is also a slight difference in intensity between chain A and chain B of the compared structures, especially the loop region of the 273_100 K DDM comparison, suggesting possible asymmetry between the two chains. Overall, the interatomic distances are underestimated because of cryocooling, and higher temperatures promote coordinated backbone rearrangement by expansion of interatomic distances.

**FIG. 6.**
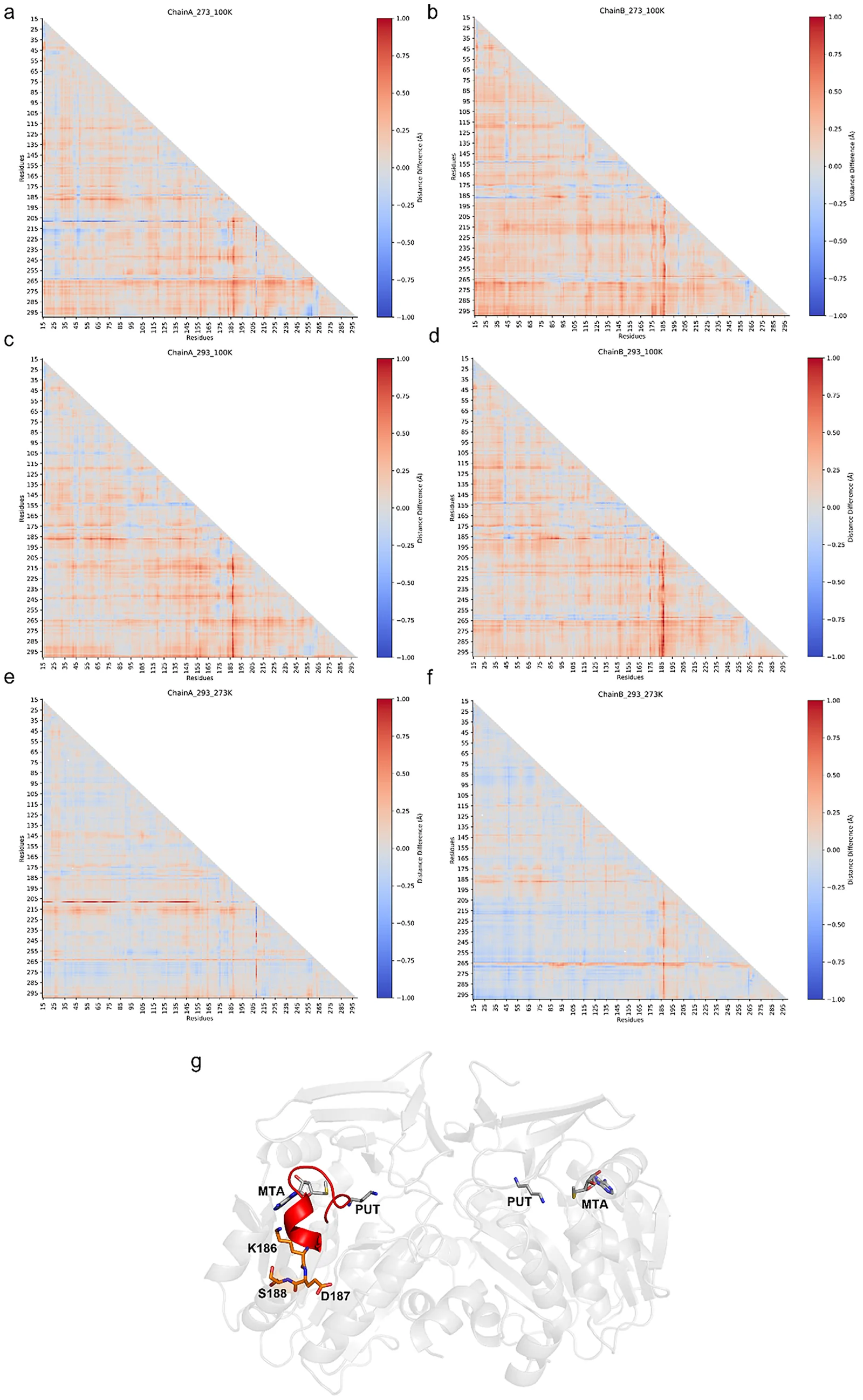
Distance difference matrix of SPDS across temperature gradients. The color scale represents distance differences (D_293/273K_–D_100K_ and D_293_–D_273K_) in angstroms (Å), with red indicating an increase in distance and blue representing a decrease in distance. (a) and (b) 293 vs 100 K comparison (c) and (d) 273 vs 100 K comparison (e) and (f) 293 vs 273 K comparison. (g) Structure of SPDS showing residues (orange) with the highest average distance differences identified by DDM. The gatekeeping loop is shown in red, and ligands are in gray.

To assess the conformational differences between the new structures and previously deposited structures, RoPE^72^ was used for torsional space comparison (Fig. S10). This analysis revealed that ambient temperature structures sample a slightly different conformational state from the cryogenic structure, as shown by the small shift in torsional space. The 273 and 293 K structures are close together, indicating that they sample similar conformational states. Crystal packing has an influence on torsional space, as the apoSPDS structure (9Q41) and the SPDS-MTA-PUT structure (2O06), with the same space group of C222_1_, sample a completely different torsional region compared to other structures with the space group of P12_1_1. The specific ligand bound to SPDS can also cause variation in torsional space. SPDS-MTA (2O05), SPDS-MTA-SPD (2O07), SPDS-dcAdoMet (2O0L), SPDS-dcSAH (3RW9), the new SPDS-PUT structure, and the 100 K SPDS-MTA-PUT structure sample different conformational space despite having the same space group, and all are cryogenic structures. The differences could also result from differences in crystal preparations because SPDS-MTA (2O05) and SPDS-MTA-SPD (2O07), with similar crystal preparations, sample similar torsional space even with different ligands bound. However, SPDS-dcAdoMet (2O0L), with the same crystal preparation as SPDS-MTA (2O05) and SPDS-MTA-SPD (2O07), samples a different torsional space from the latter two structures, suggesting the influence of specific ligand binding. Despite similar crystal preparation, SPDS-PUT also samples a distinct but similar conformational region compared to the 100 K SPDS-MTA-PUT structure, consistent with the effect of ligand composition on conformational sampling. The proximity of the two chains of SPDS structures suggests similarity in torsional space. These results demonstrate that SPDS-MTA-PUT structures collected at different temperatures sample similar torsional space, indicating that the overall protein conformation is only subtly affected by data collection temperatures. This is in contrast to AdoMetDC, a rate-limiting polyamine biosynthetic enzyme, where torsional space sampling is influenced by data collection temperatures as much as by ligand binding.^21^ RoPE analysis in the protein tyrosine phosphatase also demonstrated that high temperature induces a more global conformational state compared to high pressure, indicating that temperature can significantly influence protein conformational sampling.^81^ The separation of structures according to space group suggests that crystal packing and ligand occupancy contribute more to the observed global torsional variation than data collection temperature.

## IV. CONCLUSIONS

The structure of SPDS-PUT was collected at 100 K and solved at 1.76 Å resolution. This structure reveals PUT binding in the absence of MTA and shows that PUT alone does not stabilize the gatekeeping loop, with full stabilization observed when both ligands are bound. Multitemperature structures at 100, 273, and 293 K were determined at 1.71, 1.98, and 1.94 Å resolution, respectively. Comparison of the three structures together with previously deposited structures enabled chain-specific analysis, revealing consistently higher conformational flexibility of the gatekeeping loop in one chain relative to the other chain, suggesting that one active site is preferentially stabilized. Differences between these multitemperature structures were analyzed with Flipper and Ringer, DDM, and Rope. Flipper and Ringer reveal temperature-dependent heterogenous sampling of side chain conformations. DDM analysis reveals subtle expansion of higher temperature structures of SPDS relative to the cryogenic structure, with the largest changes localized to the helix right after the gatekeeping loop. RoPE analysis demonstrates that the observed conformational variation is influenced by crystal packing and ligand binding more than by data collection temperature. Overall, this study provides new structural insights into putrescine binding and conformational heterogeneity in SPDS.

## SUPPLEMENTARY MATERIAL

See the supplementary material for the following: Fig. S1: mechanism of catalysis by SPDS. Figure S2: active site of SPDS-PUT structure upon refinement with MTA. Figure S3: Polder omit map of PUT in the SPDS-PUT active site. Figure S4: superposition of SPDS-PUT structure with apo SPDS (9Q41) and SPDS-MTA-PUT (2O06) showing the conformation of the catalytic residue, Asp173. Figure S5: interactions of PUT with Asp173 and Asp176 and the gatekeeping loop. Figure S6: crystal packing environment of SPDS. Figure S7: Flipper peak gains across residues in 273 and 293 K relative to 100 K. Figure S8: Ringer plots of selected residues showing alternate rotameric side chains that are detectable at cryogenic temperatures. Figure S9: Ringer plots of selected residues showing alternate rotameric side chains that are detectable only at ambient temperatures. Figure S10: torsional space analysis of new SPDS structures and previously deposited human SPDS structures.

## Data Availability

The data that support the findings of this study are openly available in SBGrid at http://doi.org/10.15785/SBGRID/1264, Ref. 82; SBGrid at http://doi.org/10.15785/SBGRID/1265, Ref. 83; SBGrid at http://doi.org/10.15785/SBGRID/1266, Ref. 84; and SBGrid at http://doi.org/10.15785/SBGRID/1268, Ref. 85.
